# Pemphigus Vulgaris and Infections: A Retrospective Study on 155 Patients

**DOI:** 10.1155/2013/834295

**Published:** 2013-06-13

**Authors:** Nafiseh Esmaili, Hossein Mortazavi, Pedram Noormohammadpour, Majid Boreiri, Tahereh Soori, Iman Vasheghani Farahani, Mitra Mohit

**Affiliations:** ^1^Autoimmune Bullous Diseases Research Center, Tehran University of Medical Sciences, Tehran 1199663911, Iran; ^2^Department of Dermatology, Tehran University of Medical Sciences, Tehran 1199663911, Iran; ^3^Razi Hospital, Vahdat Islamic Square, Tehran 1199663911, Iran; ^4^Infectious Diseases Specialist, Razi Hospital, Tehran University of Medical Sciences, Tehran 1199663911, Iran; ^5^Sharif University of Technology, Tehran 1136511155, Iran; ^6^Islamic Azad University, Tehran Medical Branch, Tehran 193951495, Iran

## Abstract

*Background*. Autoimmune process and immunosuppressive therapy of pemphigus vulgaris would predispose the patients to infections. *Aim*. We aimed to study the prevalence of infection and pathogenic agents in pemphigus vulgaris patients admitted to dermatology service. *Material and methods*. This retrospective study was conducted on 155 pemphigus vulgaris patients (68 males, 87 females) admitted to dermatology service between 2009 and 2011. In this study, the diagnosis of pemphigus vulgaris was confirmed by light microscopic and direct immunofluorescence findings. Data were collected through a questionnaire. *Results*. Of 155 pemphigus vulgaris patients, 33 had infection at admission and 9 acquired nosocomial infection. In addition, 37 cases of oral candidiasis and 15 cases of localized herpes simplex were recorded. Totally, 94 cases of infection were recorded. The occurrence of infection was significantly related to the severity of disease, number of hospital admissions, and presence of diabetes mellitus. The most common pathogenic germs isolated from cultures were *Staphylococcus aureus* and *Escherichia coli*. *Conclusion*. Severity of pemphigus vulgaris and diabetes were directly related with tendency to infections. Staphylococcus aureus and Escherichia coli were the most common pathogenic agents. Due to limitations of retrospective study, a prospective study is recommended.

## 1. Introduction

Pemphigus vulgaris (PV) and pemphigus foliaceus (PF) are organ-specific autoimmune bullous diseases characterized by loss of cell adhesion (acantholysis) and blister formation [1, 2]. These dermatoses are proven to be induced by autoimmune phenomenon [1–3]. Considering this etiology, immunosuppressive therapies are the main treatments available for these disorders. Infections are important complications in these patients attributable to disruption of the epidermal barrier due to the disease itself and immunosuppression induced by treatment [4, 5].

There are many reports regarding predisposition to infections due to immunosuppressive therapy and the immunocompromised state of pemphigus patients [6, 7].

PV has a high prevalence (30 per 100,000 inhabitants) in Iran; in this regard, we have studied this autoimmune disease from many different points of view [8–10]. The aim of the present study was to determine the rate of infection and pathogenic agents in PV patients admitted to dermatology inpatients service through a retrospective study.

## 2. Material and Methods

This retrospective study was performed on 155 PV patients (87 females, 68 males) admitted to the dermatology service of Razi Hospital of Tehran in Iran, between 2009 and 2011. The mean age of patients was 41.66 ± 13.29 years (range: 14–70). Of 155 admitted PV patients, 104 patients (67.10%) were first admitted and the 51 remaining patients (32.90%) had multiple admissions.

Patients with a clinical diagnosis of PV with compatible histopathology and direct immune fluorescence (DIF) findings confirming the clinical diagnosis of PV entered the study. Light microscopic and direct immunofluorescence findings in favor of pemphigus vulgaris were suprabasal bullae and acantholysis and IgG and C3 depositions in the intercellular regions of epidermis, respectively. The severity of PV was evaluated by a “severity index for pemphigus,” namely, mild, moderate, and severe [11]. All of the PV patients contributing to this study (mild, moderate, and severe) were hospitalized in the dermatology wards of Razi Hospital regardless of severity of the disease. Accordingly, 43 patients (27.74%) had mild, 98 patients (63.23%) had moderate, and 14 patients (9.03%) had severe forms of the disease.

Regardless of the severity of pemphigus, the patients were treated with 2 mg/kg/day prednisolone and 2.5 mg/kg/day azathioprine [12]. In this study, 18 patients with mild disease with upper limit of the normal range of liver function tests (aspartate aminotransferase = 42 U/L, alanine transaminase = 41 U/L) or lower limit of the normal range of white blood cells count (4 × 10^3^/microliter or less) who were not suitable for azathioprine adjuvant therapy were treated with 2 mg/kg/day prednisolone alone. 117 patients with mild, moderate, and severe disease (mild = 25, moderate = 88, and severe = 4) were treated with 2 mg/kg/day prednisolone and 2.5 mg/kg/day azathioprine. Twenty PV patients with moderate and severe disease (moderate = 10, severe = 10) with upper limit of the normal range of liver function tests who were not suitable for azathioprine adjuvant therapy were treated with 2 mg/kg/day prednisolone and 2 g/day (4 × 500 mg/day tablet) mycophenolate mofetil [13].

The phenotype of PV recorded in order of frequency was mucocutaneous in 104 (67.10%), cutaneous in 30 (19.35%), and mucosal phenotype in 21 patients (13.55%).

Demographic data including age, gender, number of admissions, severity of the disease, underlying medical disorders such as history of diabetes and hypertension, treatment protocols, and infections recognized during the admission period were registered in the appropriate questionnaires. The ethical committee of Tehran University of Medical Sciences approved the study.

### 2.1. Statistical Analysis

Data were collected by questionnaire and analyzed by statistical software, SPSS. Chi-square test and Student's *t*-test were used for data analysis. *P* value less than 0.05 was assigned as statistically significant.

## 3. Results

In total 94 cases of infections were recorded (Table 1). Fifty two patients had a clinical diagnosis of oral candidiasis and localized oral herpes simplex. Excluding these 52 patients, 42 patients had pulmonary, bacterial skin, and urinary infections.

Excluding oral candidiasis and herpes infections, with regard to the rate of infection in men and women (20/68 versus 22/87), there was no statistically difference between the two genders.

From the 104 first admitted PV patients, 19 patients (18.27%) had infections; while from the 51 patients with multiple admissions to hospital, 23 (45.10%) had infections (*P* < 0.001) (Table 2).

With regard to 42 patients with pulmonary, bacterial skin, and urinary infections, 33 patients had infections at admission (day 0 to 2), while 9 patients were infected from day 3 and thereafter. Namely, these 9 patients had hospital-acquired infection (nosocomial infection). 

Severity of the disease is shown in Table 3. Regarding the severity of disease, the rates of infection between mild, moderate, and severe were significantly different (*P* = 0.011).

With regard to 42 patients with pulmonary, bacterial skin, and urinary infections, 16 patients had skin infection, 13 patients had urinary infections, and 13 patients had pulmonary infections (Table 1). The results of cultures of skin and urinary tract infections and percentage of resistant antibiotic to pathogenic agents are shown in Table 4.

Table 5 presents the data regarding the relationship between type of drug therapy regimen with frequency of infections. The difference between 3 immunosuppressive therapy regimens was not significant (*P* = 0.151).

Of 155 patients, 14 patients were diabetic. The rate of infection in diabetic versus nondiabetic PV patients was 50% and 24.82%, respectively (*P* = 0.044).

In PV patients with nosocomial infection, the mean duration between admission to hospital and onset of infection was 13.22 ± 5.7 day (range: 4–22). The mean dose of prednisolone at the time of nosocomial infection onset was 55.8 ± 17.9 mg/day.

No mortality was recorded in this study.

## 4. Discussion

PV is a well-known autoimmune disease [14]. Nowadays, the relationship between autoimmunity, immunodeficiency, and infection is well recognized. It is believed that autoimmunity and immunodeficiency are not separate entities, but rather some connection exists between them [15, 16]. On the other hand, hospitalization in addition to immunosuppressive therapy would predispose the PV patients to infection. 

Our search in the literature revealed some similar studies performed in other countries [6]. In our study, 60.6% of PV patients had infections, while in the study of Belgnaoui et al., 68% of patients had infections. Overall, our results are similar to the study of Belgnaoui et al. The small differences between the two studies may be due to differences in severity of disease and duration of hospitalization [6].

The study of Ljubojević et al. on 159 PV patients during 19 years revealed several complications associated with high doses of corticosteroids and immunosuppressive therapy [17]. These complications were as follows: skin infection in 26 patients (16.35%), sepsis in 9 patients (5.66%), and 14 patients (8.81%) died during the period of hospitalization. With regard to skin infection, the results of the Ljubojević et al. study are similar to those of the present study. The absence of sepsis and death in our study may be due to the small number of patients with severe PV and the shorter period of our study.

In our study, the occurrence of infection had a direct relationship with disease severity, and the difference between mild and severe was significant. In Ljubojević et al.'s study, severe cutaneous and mucosal involvement was also consistent with a higher mortality rate [17].

Mourellou et al. followed 48 patients for 11 years; they concluded that complications and mortality rate of PV were related to the severity of PV. Our study is consistent with the study by Mourellou et al. [18].

In the current study, the rate of infection in PV patients with diabetes was significantly higher than in nondiabetics (*P* = 0.044). Belgnaoui et al. also reported more severe bacterial infection in diabetics PV patients [6].

In our study, the rate of infection in patients receiving immunosuppressive adjuvants plus systemic steroids was not significantly different from patients receiving corticosteroids alone. This means that all PV patients receiving corticosteroids (with or without adjuvant immunosuppressive) are prone to infections. It should be noted that we did not include the PV patients treated with rituximab (which may be a susceptibility factor for infection). Kim et al. found that there was no difference in prednisolone alone or prednisolone plus adjuvant with regard to prognosis and time to remission in PV patients [19].

Most bacterial skin infections detected in our patients were due to *Staphylococcus aureus*. In other studies in PV patients, skin infections due to *Staphylococcus aureus* have been reported as well [20]. In the study by Kanwar and Dhar, among the causes of 10 deaths of PV, sepsis was the most common cause and the responsible pathogenic agent in 4 cases was *Staphylococcus aureus* [21].

*Escherichia coli* was the most frequent cause of urinary tract infection in our study. Obviously, *Escherichia coli* is the most common cause of urinary tract infections in the general population [22].

In the current study, 9.68% of patients had localized herpes simplex infection, while in the study of Belgnaoui et al. 17% of patients had localized herpes infection [6]. In several reports, the herpes infection has been studied in PV patients [6, 23, 24]. Although high doses of corticosteroid and immunosuppressive therapy would cause patients to be prone to an extensive herpes simplex virus infection, in this study, we had only localized herpes simplex virus infections [24]. Previously, our group had studied the herpes simplex infection and PV in Iranian patients [10]. In that study we concluded that a herpes virus infection occasionally is responsible for exacerbation of PV [10].

In the current study, 23.87% of patients had oral candidiasis, while in the study of Belgnaoui et al. 30% of patients had oral candidiasis [6]. With regard to oral candidiasis, the result of the two studies is similar. Previously, laryngeal candidiasis has been reported in patients with PV, but in this study we had only localized oral candidiasis [25].

Infection rate had a positive significant relationship with the number of admission sessions. Patients with multiple admission sessions had a rate of infection approximately two times more than patients admitted for first time. Logically, patients with a more severe disease would have more admissions, and consequently the rate of infections would increase.

Retrospective nature and relatively short period of the study (2 years) are major limitations of this project. Another limitation of the study is PV patients on different immunosuppressive adjuvant therapy included in this study. A prospective study with followup is recommended.

We concluded that PV patients with multiple admission sessions, diabetes mellitus, and severe disease are at higher risk of infection. According to a high rate of antimicrobial resistance, antibiograms are recommended for antibiotics therapy.

## Figures and Tables

**Table 1:** Prevalence of all infections in PV patients.

Diagnosis	No. of patients (% of 155)	Treatment
Oral candidiasis	37 (23.87%)	Systemic itraconazole
Herpes simplex	15 (9.68%)	Systemic Acyclovir
Skin infection	16 (10.32%)	According to pathogenic agent
Urinary bacterial infection	13 (8.39%)	According to pathogenic agent
Pulmonary infection	13 (8.39%)	Empirical regarding infectious man suggestion

All	94 (60.65%)	—

**Table 2:** Frequency of infection regarding admission of patients (excluding oral candidiasis and herpes infections).

Admission	Infection			Sum
	No infection	At admission	Nosocomial infection	
First admission	85	15	4	104
Multiple admission	28	18	5	51

**Table 3:** Frequency of infection regarding severity of PV patients (excluding oral candidiasis and herpes infections).

Severity	Infection			Total infection
	No infection	At admission	Nosocomial infection	
Mild	36	7	0	7
Moderate	71	20	7	27
Severe	6	6	2	8

**Table 4:** Pathogenic agents and percentage of resistance to antibiotics in order of frequency in skin and urinary tract infections of PV patients.

Site of involvement	Germ	Percentage	Resistant to antibiotic	Percentage
Skin			Penicillin	60%
			Cefazolin	40%
			Cephalexin	26.7%
	*Staphylococcus aureus *	15 (93.7%)	Ampicillin	20%
			Clindamycin	20%
			Vancomycin	13.3%
			Ceftizoxime	13.3%
			Cefotaxime	6.7%
	Other	1 (6.3%)	—	—

Urinary tract			Cefixime	57.1%
			Gentamicin	57.1%
			TMP	42.8%
	*Escherichia coli *	7 (53.8%)	Ampicillin	28.6%
			Amikacin	28.6%
			Ciprofloxacin	28.6%
			Cefotaxime	14.3%
			Gentamicin	100%
	*Pseudomonas*	2 (15.4%)	Cefixime	50%
			Amikacin	50%
	*Proteus*	1 (7.8%)	Ampicillin	100%
			Cefixime	100%
	Other	3 (23.0%)	TMP	66.7%
			Ampicillin	33.3%

**Table 5:** Frequency of infections regarding regimen of immunosuppressive therapy (excluding oral candidiasis and herpes infections).

Type of drug therapy	Infection			Total infection
	No infection	At admission	Nosocomial infection	
Prednisolone	14	4	0	4
Prednisolone + azathioprine	88	20	9	29
Prednisolone + mycophenolate mofetil	11	9	0	9
